# Biosynthesis of copper nanoparticles using *Solenostemma argel* and their effect on enhancing salt tolerance in barley plants

**DOI:** 10.1038/s41598-024-63641-4

**Published:** 2024-06-03

**Authors:** Hassan O. Shaikhaldein, Fahad Al-Qurainy, Mohammad Nadeem, Salim Khan, Mohamed Tarroum, Abdalrhaman M. Salih, Abdulrahman Al-Hashimi

**Affiliations:** Department of Botany and Microbiology, College of Science, King Saud University, P.O. Box 2455, 11451 Riyadh, Saudi Arabia

**Keywords:** Plant physiology, Nanoparticles

## Abstract

The distinctive characteristics of nanoparticles and their potential applications have been given considerable attention by scientists across different fields, particularly agriculture. However, there has been limited effort to assess the impact of copper nanoparticles (CuNPs) in modulating physiological and biochemical processes in response to salt-induced stress. This study aimed to synthesize CuNPs biologically using *Solenostemma argel* extract and determine their effects on morphophysiological parameters and antioxidant defense system of barley (*Hordeum vulgare*) under salt stress. The biosynthesized CuNPs were characterized by (UV–vis spectroscopy with Surface Plasmon Resonance at 320 nm, the crystalline nature of the formed NPs was verified via XRD, the FTIR recorded the presence of the functional groups, while TEM was confirmed the shape (spherical) and the sizes (9 to 18 nm) of biosynthesized CuNPs. Seeds of barley plants were grown in plastic pots and exposed to different levels of salt (0, 100 and 200 mM NaCl). Our findings revealed that the supplementation of CuNPs (0, 25 and 50 mg/L) to salinized barley significantly mitigate the negative impacts of salt stress and enhanced the plant growth-related parameters. High salinity level enhanced the oxidative damage by raising the concentrations of osmolytes (soluble protein, soluble sugar, and proline), malondialdehyde (MDA) and hydrogen peroxide (H_2_O_2_). In addition, increasing the activities of enzymatic antioxidants, total phenol, and flavonoids. Interestingly, exposing CuNPs on salt-stressed plants enhanced the plant-growth characteristics, photosynthetic pigments, and gas exchange parameters. Furthermore, CuNPs counteracted oxidative damage by lowering the accumulation of osmolytes, H_2_O_2_, MDA, total phenol, and flavonoids, while simultaneously enhancing the activities of antioxidant enzymes. In conclusion, the application of biosynthesized CuNPs presents a promising approach and sustainable strategy to enhance plant resistance to salinity stress, surpassing conventional methods in terms of environmental balance.

## Introduction

Salinity stress is a significant environmental challenge that adversely affects agricultural productivity especially in arid and semiarid regions of the world^1^. Salt stress affects approximately 50% of irrigated land and about 20% of global cropland, leading to a severe reduction in crop growth and yield^2,3^. Salinity have led to alterations in various physiological responses in plants like harming the integrity of the plasma membrane, disrupting stomatal conductivity, hinders gaseous exchange and reducing photosynthetic efficiency^4,5^. It also possess a detrimental effects by producing reactive oxygen species (ROS) including hydroxyl free radical (OH*), singlet oxygen (^1^O_2),_ and oxide ions (O_2_^−^)^6^. The accumulation of salt in the root zone creates a negative water potential, which hinders the absorption of water and the uptake of minerals by root cells^7^. Additionally, prolonged exposure to saline environments results in the accumulation of sodium ions (Na^+^) and chloride ions (Cl^−^) within cellular compartments of above-ground plant tissues, resulting in ionic toxicity^8^.

Throughout the years, several stress management strategies have been developed to enhance salinity stress tolerance in cereal crops for improving their productivity. These approaches encompass on-farm practices, screening of more resilient genotypes, introduction of genes that promote tolerance, and modification of traditional breeding methods^9,10^. However, these methods have encountered limitations due to their time-consuming, high costs, and limited adaptability. In this context, there is an increasing need for innovative and sustainable solutions to mitigate the impact of salinity stress on crops.

Nanotechnology has arisen as a versatile, effective, and widely embraced scientific method in human life, it played a crucial role in revolutionizing the field of agriculture^11,12^. Recent advancements in this field offer a promising avenue for significantly improving crop production and assuring sustainability. This potential improvement stems from the integration of nanoparticles (NPs) into strategies aimed at achieving sustainable and equitable utilization of agricultural resources^13^. Nanoparticles (NPs) are substances characterized by having a size smaller than 100 nm in at least one of their dimensions^14^. NPs exhibit significant attributes, such as a high surface area relative to their volume, diminutive size, shape adjustability, and the ability to effectively transport various substances to cells; thus, they are currently under examination for their potential applications in agriculture as nanofertilizers, nanopesticides, and carriers for a variety of plant growth regulators^15,16^. Furthermore, utilizing nanoparticles represents a novel approach to enhance plant growth and performance in saline conditions^17^. Many recent studies have highlighted the capability of NPs to act as agents that alleviate abiotic stress and promote the growth and development of plants^18,19^. This is mainly linked to their diminutive dimensions and their efficiency in transporting minerals and chemicals at the cellular level^20^. Singh et al.^21^ conducted a study involving the use of ZnO NPs, their findings revealed improved agronomic traits and increased antioxidant enzyme activities in rice (*Oryza sativa*) when exposed to salinity conditions. Additionally, the application of calcium nanoparticles to tomato plants was found to enhance plant defense mechanisms against salt stress by lessening the oxidative damage to membranes and maintaining balance in ionic state^22^.

Among the various nanoparticles under investigation, copper nanoparticles (CuNPs) have gained attention for their multifaceted properties and biocompatibility with plants^23^. Copper nanoparticles have been synthesized using various methods, including chemical, physical, and biological approaches^24^. The biological synthesis of nanoparticles, using plant materials offers biocompatibility and eco-friendly alternative to conventional chemical methods^25,26^. Where, many studies have documented the eco-friendly creation of copper nanoparticles through various plant extracts like *Calotropis gigantea*^27,28^. Such biosynthesized nanoparticles exhibit distinctive traits that render them well-suited for mitigating salinity stress in crops^29^. Copper plays a vital role as a fundamental element in numerous enzymes that are essential for redox and electron transfer processes within plant cells, thus it serves as a significant micronutrient capable of enhancing both growth and various developmental stages^30^. Reckoning this significant fact, CuNPs have been developed and examined for their ability to promote growth, improve development, manage diseases, and induce drought tolerance in plants^31–33^. To our knowledge, there have no comprehensive studies been conducted to assess the physiological and biochemical responses of barley plants to copper nanoparticles application in saline conditions.

Barley (*Hordeum vulgare*) is an important cereal crop that is often grown in regions prone to salinity stress for forage purposes and as a grain crop. The crop is known for its adaptability and resilience, yet its growth and yield can be significantly hampered by high soil salinity^34^. Where the susceptibility of the crop to salinity is most pronounced during the germination and early seedling stages^35^. The purpose of this study was to examine the potential impacts of biosynthesized copper nanoparticles in enhancing salinity stress tolerance in barley.

## Material and methods

### Synthesis of CuNPs

*Solenostemma argel*, a medicinal plant collected from botanical garden of Department of Botany, King Saud University, is commonly known as argel and thrives in desert environments. Previous studies, supported by GC–MS analysis, have revealed a plethora of phytochemicals within the plant^36,37^. These phytochemical constituents play a crucial role in the NPs formation as they act as reducing agents in the biological synthesis of various nanoparticles.

In this study, copper nanoparticles were synthesized in a green approach using *Solenostemma argel*. Copper (II) sulfate pentahydrate (CuSO_4_⋅5H_2_O) was purchased from Sigma-Aldrich Chemical Corp. All the solutions were made using deionized Milli-Q water. In the typical synthesis of copper nanoparticles, 5 g of dried leaves of *S. argel* were meticulously cleaned, then transferred in a round-bottom flask with 100 mL of deionized water. The mixture was boiled for 10 min, and the resulting aqueous extract was filtered and stored in a refrigerator at 4 °C for further use. 50 ml of the leaf extract was added into 100 ml of 1 mM of copper sulfate solution and kept in stirring for 24 h at room temperature. The color of the copper sulfate aqueous solution changed from blue to dark green confirming the initial formation of the copper nanoparticles synthesis.

### Characterization techniques of synthesized CuNPs

The characterization of green-synthesized Cu nanoparticles was analyzed by different techniques. First, the surface plasmon resonance (SPR) band for the synthesized NPs was identified via ultraviolet–visible (UV–Vis) spectroscopy analysis. The presence of potential biomolecules and functional groups that acted as reduction agents for CuNPs synthesis were monitored using Fourier transform infrared spectroscopy (FT-IR). The crystalline behavior of the biosynthesized CuNPs was investigated using the X-ray diffraction method (XRD). The transmission electron microscope (SEM) was used to identify the size and shape of the biosynthesized nanoparticles.

### Plant material and growth conditions

Seeds of barley (*Hordeum vulgare*) were surface sterilized with 0.1% sodium hypochlorite (NaOCl) solution for 15 min and then thoroughly washed with sterile distilled water under aseptic conditions before being used in the experiments.

The study was performed in controlled conditions at the Botany and Microbiology Department of King Saud University as a factorial experiment in a completely randomized design (CRD). The experiment consisted of nine treatments, three levels of salinity (0, 100, and 200 mM NaCl) as well as three levels of CuNPs (0, 25, and 50 mg/L). Each treatment was implemented in triplicates. The sterile barley seeds were planted in a plastic pot. Each pod contained 800 g of soil as well as ten seeds of *H. vulgare*. The plants were collected 25 days post-sowing for growth and physiological assessments.

### Measurement of growth parameters

The roots and shoots of plants uprooted at 25 days of age were washed with deionized water. Subsequently, the plant tissues soaked in water were dried on Whatman filter paper before measuring growth parameters. The lengths of plant roots and shoots were measured using a measuring scale, and the samples’ fresh weight was then determined using a sensitive electronic balance. To determine the dry weights of roots and shoots, the samples were subjected to oven-drying at 80 °C for a duration of 48 h. The leaf area index was assessed using a portable leaf area meter (ADC Bioscientific, UK). The diameter of the stem was measured in the middle internode of the barley stem using a Vernier caliper.

### Gas exchange parameters

The gas exchange parameters such as net photosynthetic rate (*Pn*), stomatal conductance (*gs*), transpiration rate (*Tr*), and internal CO_2_ (*Ci*) content were calculated via infrared portable photosynthetic system (LI-COR 6400, LICOR, Lincoln, Nebraska, USA). All the parameters are determined in the intact plant leaves at room temperature and relative humidity (60%). Photosynthetic photo-flux density (PPFD) and CO_2_ concentration were kept constant at 800 μmol mol^−2^ s^−1^ and 600 ppm, respectively.

### Biochemical parameters

#### Determination of chlorophyll and carotenoid contents

The amounts of photosynthetic pigments were estimated using the method reported by Arnon^38^. Fresh leaves of barley (100 mg) were crushed in 10 ml of chilled 80% acetone using pre-chilled mortar and pestle to obtain a fine pulp. The pulp was centrifuged in a High-Speed Refrigerated Microcentrifuge (M1324R, USA) at 1000*g*, at 4 °C for 10 min. The absorbance of the supernatant was measured against 80% acetone as blank at 470, 645 and 663 nm using UV-1800 spectrophotometer (Shimadzu, Japan) for the calculation of chlorophyll and carotenoid contents. The chlorophyll a and b, and carotenoid contents were calculated as follows.

$$chlorophyll \; a \left(Ch \; a\right)=12.7 \times \text{A}663- 2.69\times \text{A}645$$

$$chlorophyll \; b \left(Ch \; b\right)=22.9 \times \text{A}645- 4.68\times \text{A}663$$

$$Carotenoids =\frac{1000 \times \text{ A}470-3.27 \times Ch \; a-104 \times Ch \; b}{229}$$

where A663, A645, and A470 are the absorbance value read at 663 nm, 645 nm, and 470 nm, respectively.

#### Electrolyte leakage (EL) measurement

Electrolyte Leakage was calculated as per method described by Bajji et al.^39^. Removal of dust and contamination that occurred during sampling was performed by a slow wash of the leaves using deionized water. The leaf blade samples were placed in a test tube containing 20 mL of deionized water at room temperature, then after, an electrical conductivity meter (DDSJ-308A, Shanghai) was used to determine the electrical conductivity (E0) of the solution immediately. Following a day of incubation at 35 °C, the electrical conductivity (E1) was read again. The tubes were then flooded in a 120 °C water bath for 30 min and cooled to 25 °C before recalculating the electrical conductivity (E2). Finally, the electrolyte leakage percent (EL %) of the leaf cells was measured as follows:

$$\text{Electrolyte Leakage }(\text{EL\% })=\frac{\text{E}1 -\text{ E}0}{\text{E}2 -\text{ E}0} \times 100$$

#### Total protein and soluble sugars contents determination

Soluble sugars were extracted using the phenol sulfuric acid technique^40^. In this method, 300 mg of plant tissue was immersed in ethanol (10 mL). The extract at 10,000 rpm for 10 min was centrifuged and then treated with 5% phenol and 98% H_2_SO_4_. The mixture was subjected to a boiling water bath for 30 min and then cooled. The absorbance was measured at 490 nm using UV–Vis spectroscopy. A glucose standard curve was used to estimate the concentration of the total soluble sugars.

#### Lipid peroxidation and proline assay

The level of MDA concentration in fresh leaves was estimated according to Jiang and Zhang^41^ method. Fresh leaf (0.4 g) was homogenized in 5 mL of 0.1% (w/v) trichloroacetic acid, and the homogenate was centrifuged at 7000 rpm for 10 min. Then, 1 mL of the supernatant was mixed with 4 mL of 0.5% thiobarbituric acid (TBA, in 20% TCA), and the blend was heated to 95 °C for half an hour. Then, the mixture immediately was placed in an ice bath for cooling and centrifuged at 7000 rpm for 10 min; the supernatant absorbance (containing MDA) was read against a reagent blank (0.5% TBA in 20% (w/v) TCA) at 532 nm and corrected to non-specific turbidity by subtracting the value at 600 nm on a UV–1800 spectroscopy.

Bates et al.^42^ procedure was used to estimate the proline levels in barley leaf. Fresh barley leaves (0.5 g) were crushed in 10 mL of 3% sulfosalicylic acid and centrifuged at 10,000*g* for 15 min. Subsequently, the 2 mL supernatant was taken in a test tube and combined with 2 mL of ninhydrin reagent and glacial acetic. Afterward, the samples were placed in a water path at 100 °C for 30 min before incubating the mixture in an ice bath for 5 min to end the reaction. Then, 5 mL of toluene was added and vigorously mixed and stand for 15 min at room temperature. The absorbance of the developed color in the upper phase at 520 nm was measured using a UV–Vis spectroscopy and toluene was used as a blank.

#### Quantitation of hydrogen peroxide (H_2_O_2_)

Hydrogen peroxide (H_2_O_2_) level was determined as per Velikova et al.^43^ method. A known weight of barley fresh leaves was ground in 5 mL of 0.1% (w/v) trichloroacetic acid (TCA). The homogenate was centrifuge at 7000*g* for 30 min; thereafter, 0.5 mL of the supernatant was blended with 0.5 mL of 10 mM potassium phosphate buffer (pH 7.2) and 1.0 mL of 1.0 M KI solution. The absorbance was read via spectrophotometer at 390 nm.

### Estimation of antioxidant enzyme activities

Different parts of plant samples (Shoot and Roots) were initially blended in liquid nitrogen and dissolved in 100 mM sodium phosphate buffer (pH 7.2) for further sample preparation according to the specific protocols listed below. After centrifugation at 7000×*g* for 15 min at 4 °C, the upper phase was placed in a falcon tube and used to estimate the activity of enzymatic antioxidants. The protein content in the mixture was determined as per method described by Bradford^44^.

#### Superoxide dismutase (EC 1.15.1.1)

The activity of superoxide dismutase (SOD) was determined according to Marklund and Marklund^45^ procedure’s. The reaction mixture consisted of 1.9 mL 100 mM sodium phosphate buffer (pH 7.2), 0.25 mM pyrogallol, and 100 μL of plant extract. The absorbance was recorded at 420 nm. The SOD activity (U g^−1^ protein) was defined as the amount of enzyme that inhibits 50% of pyrogallol oxidation.

#### Catalase (EC 1.11.1.6)

The catalase (CAT) activity was measured spectrophotometrically by recording the absorbance at 240 nm using Claiborne^46^ method’s. The reaction mixture contained 1 mL of 0.059 M H_2_O_2_ in 0.1 M sodium phosphate buffer (pH 7.2), 1.8 mL of distilled water, and 200 μL of plant extract. The CAT activity was expressed as unit g^−1^ of protein.

#### Glutathione reductase (EC 1.6.4.2)

The activity of glutathione reductase (GR) enzyme was assessed as per protocol reported by Schaedle and Bassham^47^. The homogenate was followed oxidation of nicotinamide adenine dinucleotide phosphate (NADPH) and rea via spectrophotometer at 340 nm (e, 6.2 mM_1 cm_1) for 3 min in 2 mL of an assay mixture consists of 3 mM Na_2_EDTA, 50 mM potassium-phosphate buffer (pH 7.2), 0.15 mM NADPH, 0.5 mM GSSG, and 100 μL of plant extract. The glutathione reductase activity was expressed as EU mg^−1^ protein.

#### Ascorbate peroxidase (EC 1.11.1.11)

Ascorbate peroxidase (APX) activity was assessed by reducing the ascorbate content as described by Nakano and Asada^48^. The reaction mixture contained 0.5 mM ascorbic acid, 2 mL of 50 mM sodium phosphate buffer (pH 7.2), 0.1 mM EDTA, 0.1 mM H_2_O_2_, and 100 μL of plant extract. The absorbance of the mixture was read spectrophotometry at 290 nm for 180 s. The activity of APX was expressed as unit g^−1^ of protein.

### Estimation of phenolic and flavonoid contents

The total phenolic content (TPC) in barley leaves was estimated using The Folin–Ciocalteu reagent in accordance with the method described by Velioglu et al.^49^. A hundred milligram of fresh leaf was digested in 99% Ethanol. The samples were subjected to an orbital shaker at 200 rpm for 2 h. The mixtures were centrifuged for 5 min at 10,000 rpm and the supernatant was poured into a 2 mL Eppendorf tube. Then, 100 μL of the extract was mixed with 0.75 mL of Folin–Ciocalteu reagent in a test tube. The mixture was allowed to stand for 5 min at room temperature, and then 0.75 mL of Na_2_CO_3_ solution was added to the mixture. After 30 min of incubation at room temperature, the absorbance was read at 765 nm via a UV-1800 spectrophotometer (Shimadzu, Kyoto, Japan). The Gallic acid was subjected to plot the standard calibration curve and phenolic content was expressed in Gallic acid equivalent. For total flavonoids content (TFC), 20 μL plant extract, 0.16 mL di-ionized water, 10 μL CH_3_CO_2_K, and 10 μL AlCl_3,_ were thoroughly jumbled and then stood for 15 min. Gallic acid was used to plot the standard calibration curve. TPC content was detected as mg/g FW at 420 nm using a (UV–Vis) spectroscopy.

### Statistical analysis

The data was analyzed statistically using a two-way analysis of variance (ANOVA) and mean separation between treatments was performed using Duncan’s new multiple range test with a significance level set at (p ≤ 0.05). The entire analysis was performed using SPSS v. 20 for Windows. Principal component analysis (PCA) and Pearson's correlation analysis were conducted using Origin Pro software, version 2023.

### Ethical approval

The authors confirm that all materials/methods used in this study comply with relevant institutional, national, and international guidelines legislation.

## Results

### Green synthesis and characterization of CuO nanoparticle

#### Visual identification

**Figure 1:**
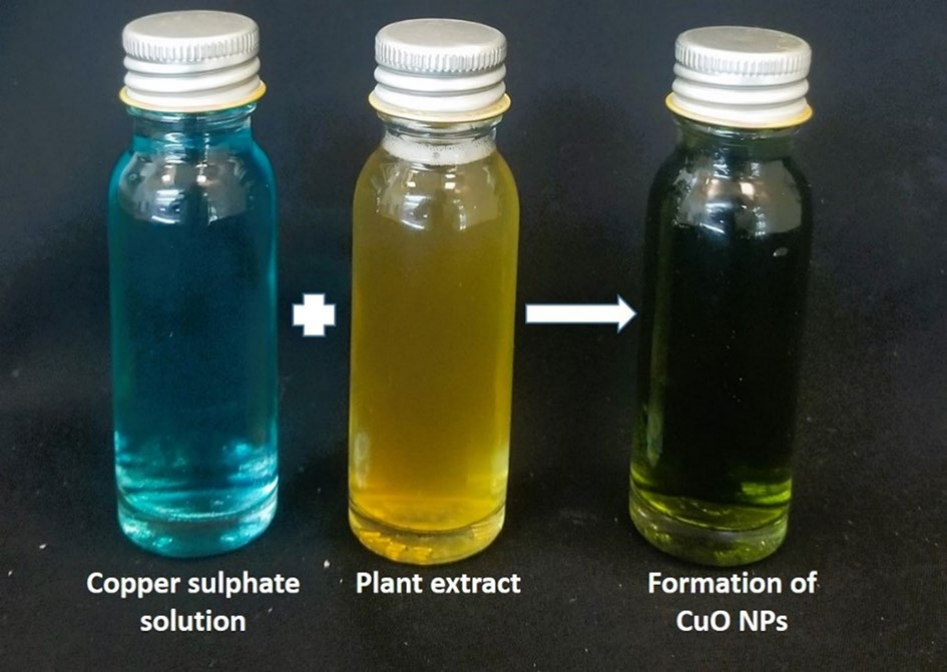
Photographic images of the synthesized CuNPs.

During the synthesis process of CuNPs, a different color array appeared. Where the extract of *S. argel* plant consists of several phytochemicals that act as reducing agents and converts the copper sulfate into copper nanoparticles. The biosynthesized CuNPs were identified visually when the color of the reaction mixture turned to dark green within 24h of stirring confirming the initial formation of CuNPs (Fig. 1).

#### UV–Vis spectroscopic analysis of copper nanoparticles

**Figure 2:**
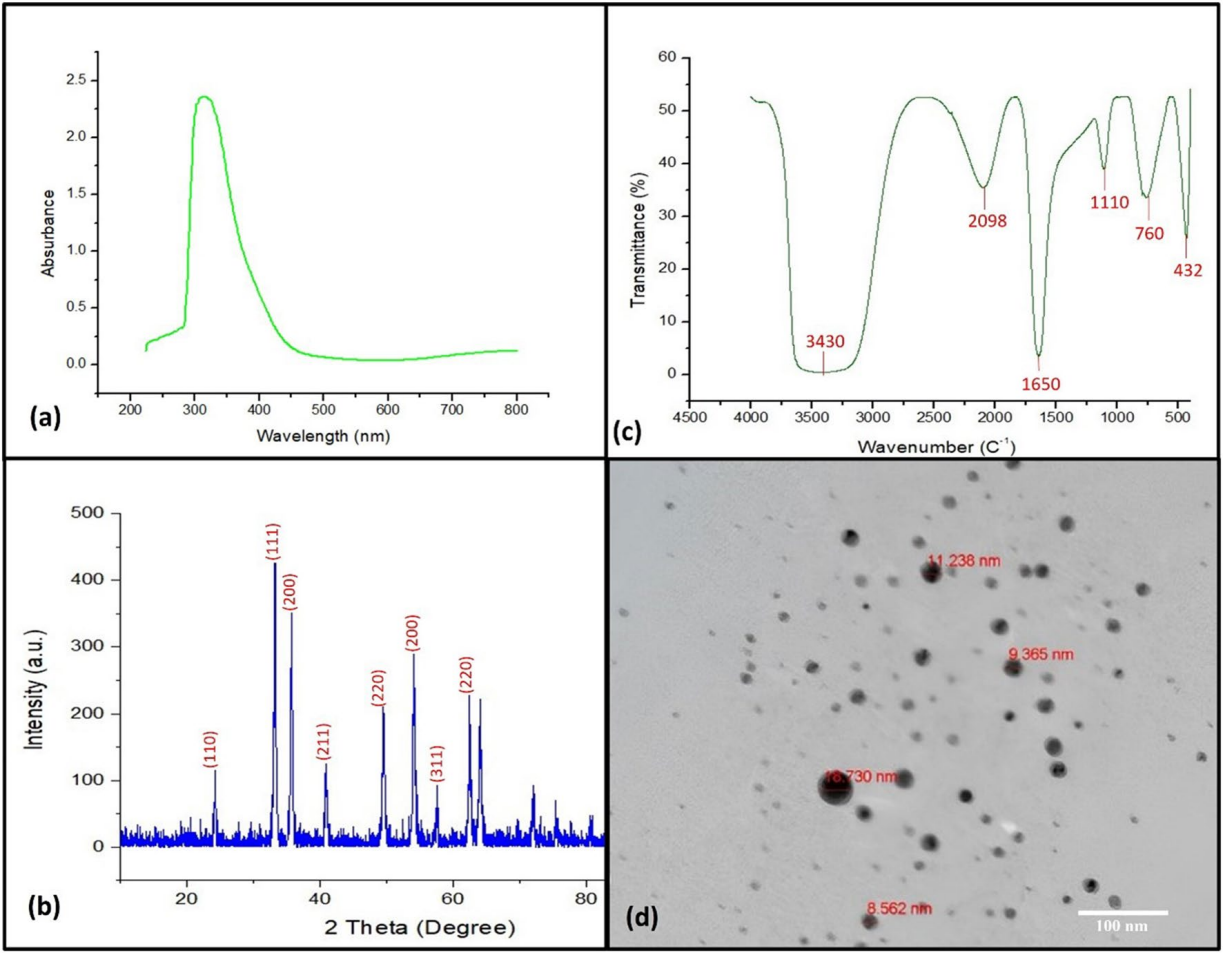
Characterization of biosynthesized CuNPs from *S. argel* leaf extract, (**a**) Ultraviolet–visible absorption spectrum, (**b**) XRD patterns, (**c**) Fourier-transform infrared spectroscopy (FTIR), and (**d**) transmission electron microscope (TEM).

The surface plasmon resonance characteristic of the bio-fabricated CuNPs were observed via UV–Vis spectroscopy at a different wavelength from 200 to 800 nm as shown in Fig. 2a. The UV–Vis spectrum analysis illustrated ideal absorption peak at 320 nm.

#### XRD analysis

The successful synthesis of copper nanoparticles was validated by examining the X-Ray diffraction. Bragg’s reflection analysis of copper nanoparticles revealed distinctive diffraction peaks at approximately 2θ = 38.34°, 43.4°, 51.3°, 73.39° and 78.36°. These peaks correspond to the crystallographic planes [110], [111], [200], [220] and [311], respectively, indicating the face-centered cubic (fcc) structure of the nanoparticles, in comparison to the standard powder diffraction card from JCPDS, specifically copper file No. 01–078-2076 (Fig. 2b).

#### FT-IR spectral analysis

The functional groups of the biosynthesized CuNPs due to chemical interaction between *S. Argel* leaf extract and Cu^2+^ were predicted using FTIR spectra. The FT-IR analysis revealed six peaks at 432 cm^−1^, 760 cm^−1^, 1110 cm^−1^, 1650 cm^−1^, 2098 cm^−1^, and 3440 cm^−1^ (Fig. 2c). The shift in the peaks was clearly attributed to the reduction of Cu^2+^ into CuNPs.

#### Transmission electron microscope (TEM) analysis

Transmission electron microscope (TEM) analysis of copper nanoparticles biosynthesized from the leaf extract of *S. argel* revealed spherical particles with sizes below 19 nm, as depicted in Fig. 2d.

### Barley growth and development

**Figure 3:**
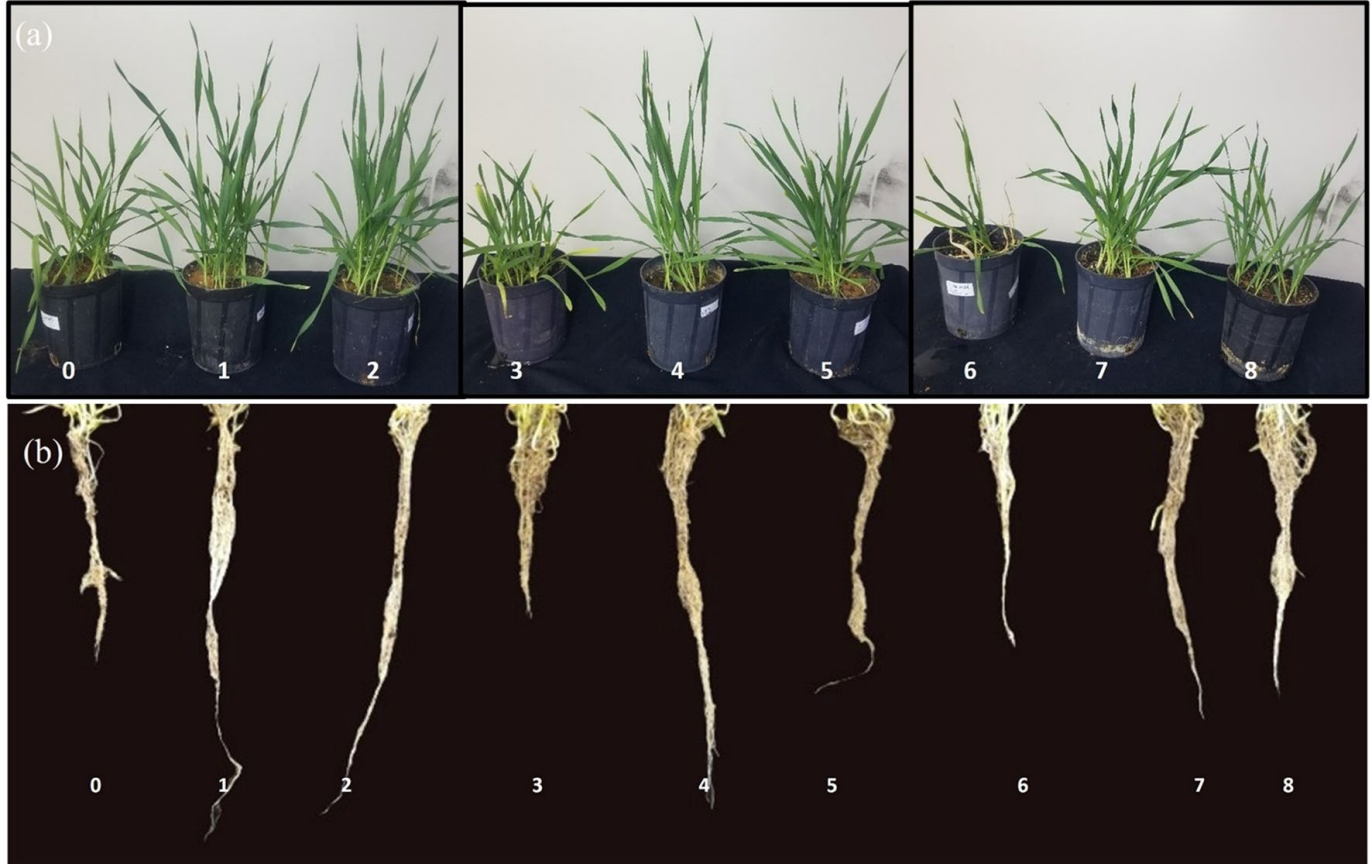
Barley (*H. vulgare*) plants under NaCl stress and application of biosynthesized silver nanoparticles. (**a**) (shoots), (**b**) roots. Where, 0 (control), 1 (25 mg/L NPs), 2 (50 mg/L NPs), 3 (100 mM NaCl), 4 (25 mg/L NPs + 100 mM NaCl), 5 (50 mg/L NPs + 100 mM NaCl), 6 (100 mM NaCl), 7 (25 mg/L NPs + 200 mM NaCl), 8 (50 mg/L NPs + 200 mM NaCl).

The application of NaCl (100 and 200 mM) salt to barley plants caused a remarkable suppression in the fresh weight, dry weight, and plant length for both shoots and roots, in addition it reduced leaf area index and stem dimeter compared to control plants. On the other hand, it caused a significant increase in EL over the control (Table 1). However, the application of Cu NPs to salt-stressed barley plants successfully alleviates the mischievous affects symptoms caused by the NaCl stress through increasing all morphological attributes (Fig. 3). While significantly reduced the EL.

**Table 1:** The impact of CuNPs and salt-induced stress, whether applied individually or in conjunction with each other on the gas exchange parameters in barley (*H. vulgare*).

NaCl	CuNPs	Fresh weight (g)		Dry weight (g)		Plant height (cm)		LA(mm)	SD (mm)	EL (%)
		Leaf	Root	Leaf	Root	Leaf	Root			
0 NaCl	0 mg/L NPs	10.8 ± 0.2^c^	4.4 ± 0.3^d^	3.7 ± 0.15	1.5 ± 0.01	23.1 ± 0.3^e^	22.7 ± 0.4^d^	70.2 ± 1.3^c^	10.2 ± 0.1^d^	14.2 ± 1.3^e^
	25 mg/L NPs	14.9 ± 0.2^a^	7.4 ± 0.2^a^	5.9 ± 0.15	2.5 ± 0.02	36 ± 0.5^a^	41.8 ± 1.0^a^	84.6 ± 0.6^a^	24.7 ± 0.3^a^	17.8 ± 0.6^de^
	50 mg/L NPs	14.0 ± 0.3^ab^	7.0 ± 0.2^a^	5.7 ± 0.1	2.6 ± 01	35.5 ± 0.4^a^	40.7 ± 0.5^a^	80.9 ± 0.8^a^	24.5 ± 0.2^a^	18.6 ± 0.8^d^
10 NaCl	0 mg/L NPs	7.5 ± 0.40^d^	3.3 ± 0.3^e^	3.0 ± 0.1	1.4 ± 0.01	17.1 ± 0.4^d^	17.8 ± 0.3^e^	52.5 ± 1.4^d^	9.3 ± 0.2^de^	27.0 ± 1.4^a^
	25 mg/L NPs	13.7 ± 0.6^ab^	6.7 ± 0.3^a^	5.2 ± 0.2	2.3 ± 01	31.2 ± 0.3^b^	32.1 ± 0.3^b^	79.8 ± 0.8^b^	22.3 ± 0.2^b^	17.8 ± 0.8^d^
	50 mg/L NPs	13.6 ± 0.4^ab^	5.4 ± 0.4^c^	4.9 ± 0.1	2.2 ± 01	29.4 ± 0.3^c^	29.1 ± 0.3^c^	76.2 ± 1.1^b^	18.6 ± 0.2^c^	19.2 ± 1.1^bc^
200 NaCl	0 mg/L NPs	6.1 ± 0.45^e^	2.8 ± 0.4^f^	2.6 ± 0.02	1.2 ± 0.02	15.6 ± 0.40^f^	15.1 ± 0.3^f^	43.9 ± 0.8^e^	8.4 ± 0.3^e^	29.9 ± 0.8^a^
	25 mg/L NPs	11.1 ± 0.35^b^	6.3 ± 0.3^b^	4.5 ± 0.1	1.9 ± 0.02	28.8 ± 0.4^c^	29.6 ± 0.3^b^	71 ± 1.0^c^	17.5 ± 0.2^c^	18.9 ± 1.0^c^
	50 mg/L NPs	10.4 ± 0.4^c^	4.6 ± 0.3^d^	4. 3 ± 0.15	1.8 ± 01	25.1 ± 0.4^d^	26.3 ± 0.3^e^	68.6 ± 1.6^c^	18.2 ± 0.3^c^	21.0 ± 1.6^b^

All the data are means of three replicates ± standard deviation. Different letters indicate significant differences between treatments according to Duncan’s multiple range test (p ≤ 0.05).

### Gas exchange parameters

As shown in Table 2, all the gas exchange parameters including net photosynthetic rate (*Pn*), intracellular CO_2_ concentration (*Ci*), transpiration rate (*Tr*), and stomatal conductance (*gs)* were considerably decreased in salt-treated barley, the highest decreased was recorded in 200 mM NaCl compared to control. Application of copper nanoparticles significantly affected leaf gas exchange attributes under NaCl stress conditions. Treatment of 25 and 50 mg/L CuNPs to stressed plants resulted in a considerable increase in the values of gaseous exchange parameters under salt stress. where, treatment of 25 mg/L NPs alone, yielded the highest values for all leaf gas exchange parameters.

**Table 2:** The impact of CuNPs and salt-induced stress, whether applied individually or in conjunction with each other on the gas exchange parameters in barley (*H. vulgare*).

NaCl	CuNPs	*P*_*n*_ (μmol m^−2^ s^−^)	*Ci* (μmol mol^−1^)	*Tr* (μmol m^−2^ s^−^)	*g*_*s*_ (μmol m^−2^ s^−^)
0 NaCl	0 mg/L NPs	1.5 ± 0.05^d^	722.3 ± 1.2^e^	0.44 ± 0.02^d^	0.09 ± 0.002^e^
	25 mg/L NPs	3.1 ± 0.1^a^	793.7 ± 2.4^a^	0.64 ± 0.04^a^	0.113 ± 0.002^b^
	50 mg/L NPs	3.0 ± 0.1^a^	753.8 ± 2.3^b^	0.58 ± 0.02^b^	0.115 ± 0.003^a^
100 NaCl	0 mg/L NPs	1.3 ± 0.05^e^	715.6 ± 2.1^f^	0.32 ± 0.03^e^	0.072 ± 0.002^f^
	25 mg/L NPs	2.6 ± 0.1^b^	743.2 ± 1.3^c^	0.57 ± 0.01^b^	0.106 ± 0.002^c^
	25 mg/L NPs	2.3 ± 0.1^c^	726.6 ± 2.1^d^	0.52 ± 0.03^c^	0.104 ± 0.002^c^
200 NaCl	0 mg/L NPs	1.0 ± 0.1^f^	704.2 ± 2.3^g^	0.28 ± 0.02^f^	0.058 ± 0.002^g^
	25 mg/L NPs	1.6 ± 0.15^d^	728.4 ± 2.0^d^	0.46 ± 0.03^d^	0.098 ± 0.001^d^
	50 mg/L NPs	1.5 ± 0.1^d^	727.7 ± 1.3^d^	0.44 ± 0.05^d^	0.095 ± 0.002^d^

All the data are means of three replicates ± standard deviation. Different letters indicate significant differences between treatments according to Duncan’s multiple range test (p ≤ 0.05).

### Photosynthetic pigments contents

**Figure 4:**
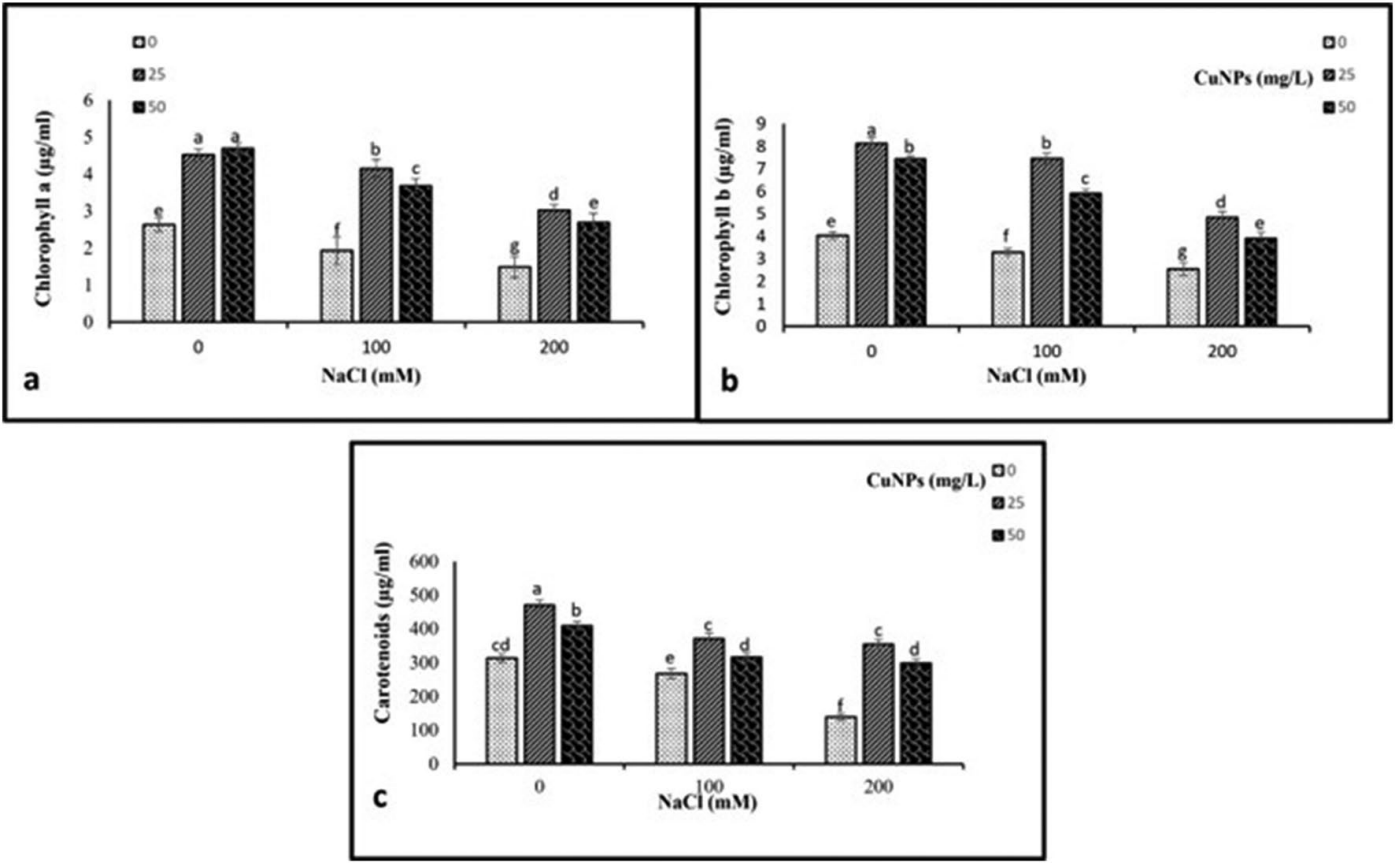
The impact of CuNPs and salt-induced stress, whether applied individually or in conjunction with each other on the photosynthetic pigments contents, (**a**) *Ch a*, (**b**) *Ch b*, and (**c**) carotenoids in barley (*H. vulgare*). All the data are means of three replicates ± standard deviation. Different letters indicate significant differences between treatments according to Duncan’s multiple range test (p ≤ 0.05).

Salt stress provoked a significant reduction in photosynthetic pigments including chlorophyll a, chlorophyll b and carotenoids comparing to control group. The most significant reduction was observed in plants subjected to 200 mM NaCl treatment. Nevertheless, the application of CuNPs, either alone or in combination with NaCl, predominantly improved photosynthetic pigments in both unstressed and stressed barley plants (Fig. 4).

### Soluble proteins, soluble sugars, and proline contents

**Figure 5:**
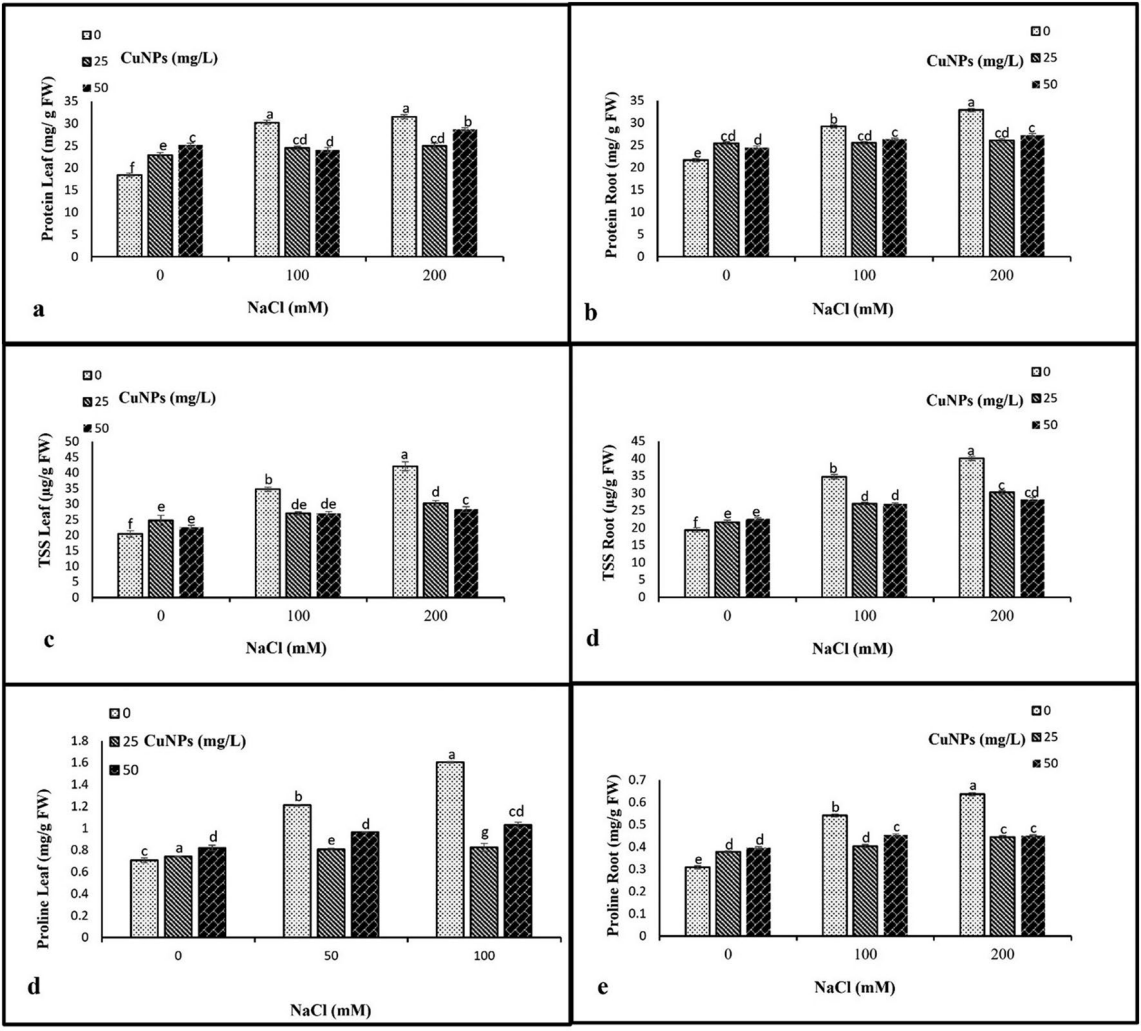
The impact of CuNPs and salt-induced stress, whether applied individually or in conjunction with each other on the osmolytes contents, (**a**) soluble proteins leaf, (**b**) soluble proteins root, (**c**) soluble sugars leaf, (**d**) soluble sugars root, (**e**) proline leaf, and (**f**) proline root in barley (*H. vulgare*). All the data are means of three replicates ± standard deviation. Different letters indicate significant differences between treatments according to Duncan’s multiple range test (p ≤ 0.05).

Exposure of barley plants to salinity stress led to a significant accumulation of osmolytes including soluble proteins, soluble sugar, and proline contents. The plants exposed to 200 mM salt without CuNPs application recorded the highest levels in soluble proteins, soluble sugar, and proline for both leaves and roots. The application of CuNPs in its all concentrations (25 and 50 mg/L) successfully overcame the harmful effect of salt stress and caused a remarkable reduction in all osmoregulatory substances compared to only salt-stressed plants (Fig. 5).

### Hydrogen peroxide (H_2_O_2_) and lipid peroxidation analyses (MDA)

**Figure 6:**
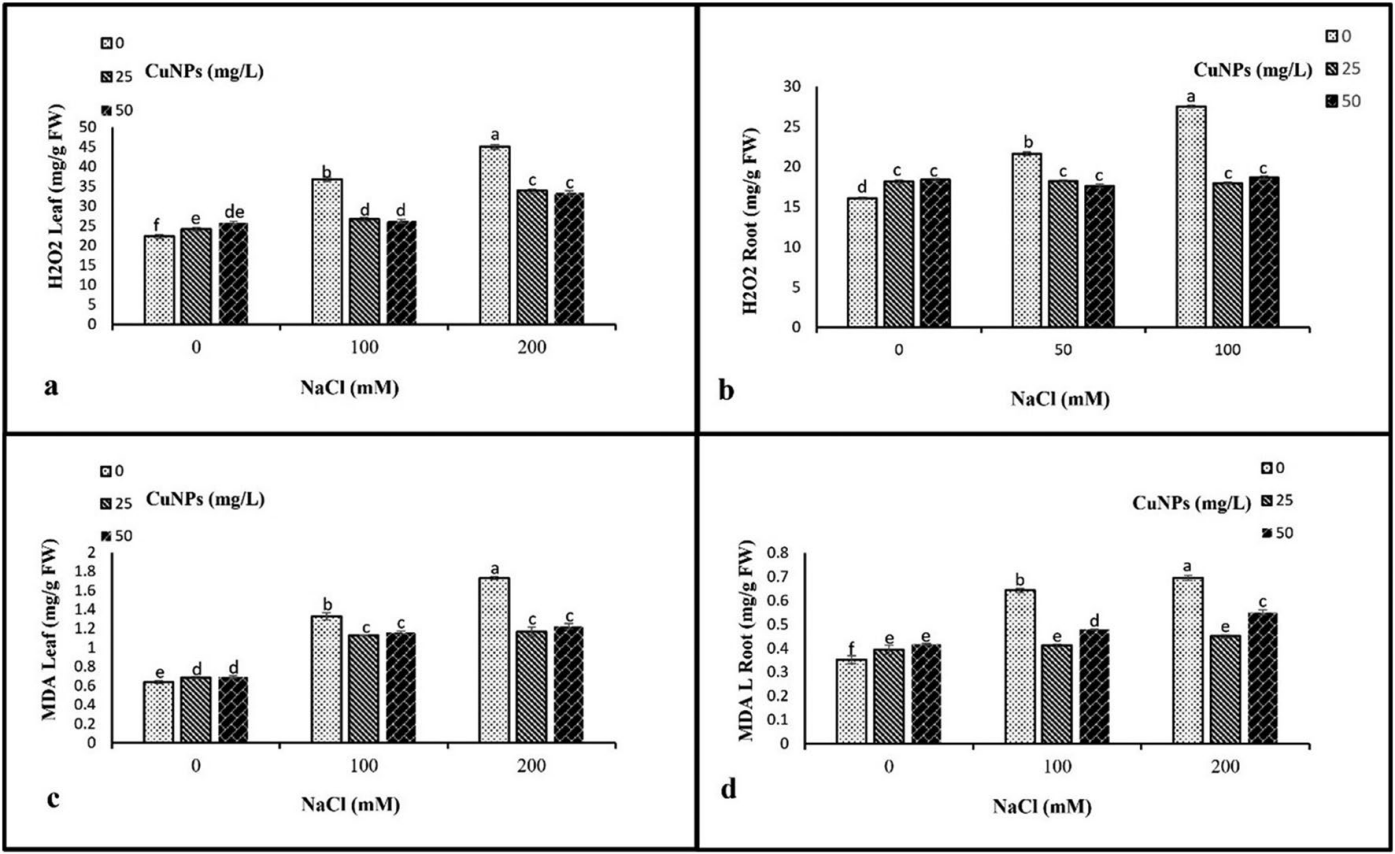
The impact of CuNPs and salt-induced stress, whether applied individually or in conjunction with each other on the hydrogen peroxide (H_2_O_2_) and lipid peroxidation analyses (MDA), (**a**) H_2_O_2_ leaf, (**b**) H_2_O_2_ root, (**c**) MDA leaf, (**d**) MDA root in barley (*H. vulgare*). All the data are means of three replicates ± standard deviation. Different letters indicate significant differences between treatments according to Duncan’s multiple range test (p ≤ 0.05).

Our results indicate that the application of only salinity treatments (100 and 200 mM) significantly increased the levels of H_2_O_2_ in leaves, as well as in roots compared to the control barley. The introduction of CuNPs to salt-stressed barley plants led to a notable decrease in MDA and H_2_O_2_ levels in both leaves and roots. The most significant reduction was observed in plants treated with 25 mg/L of CuNPs supplementation under 100 and 200 mM NaCl stress, as compared to their respective control treatments (Fig. 6).

### Antioxidant enzymes activities

**Figure 7:**
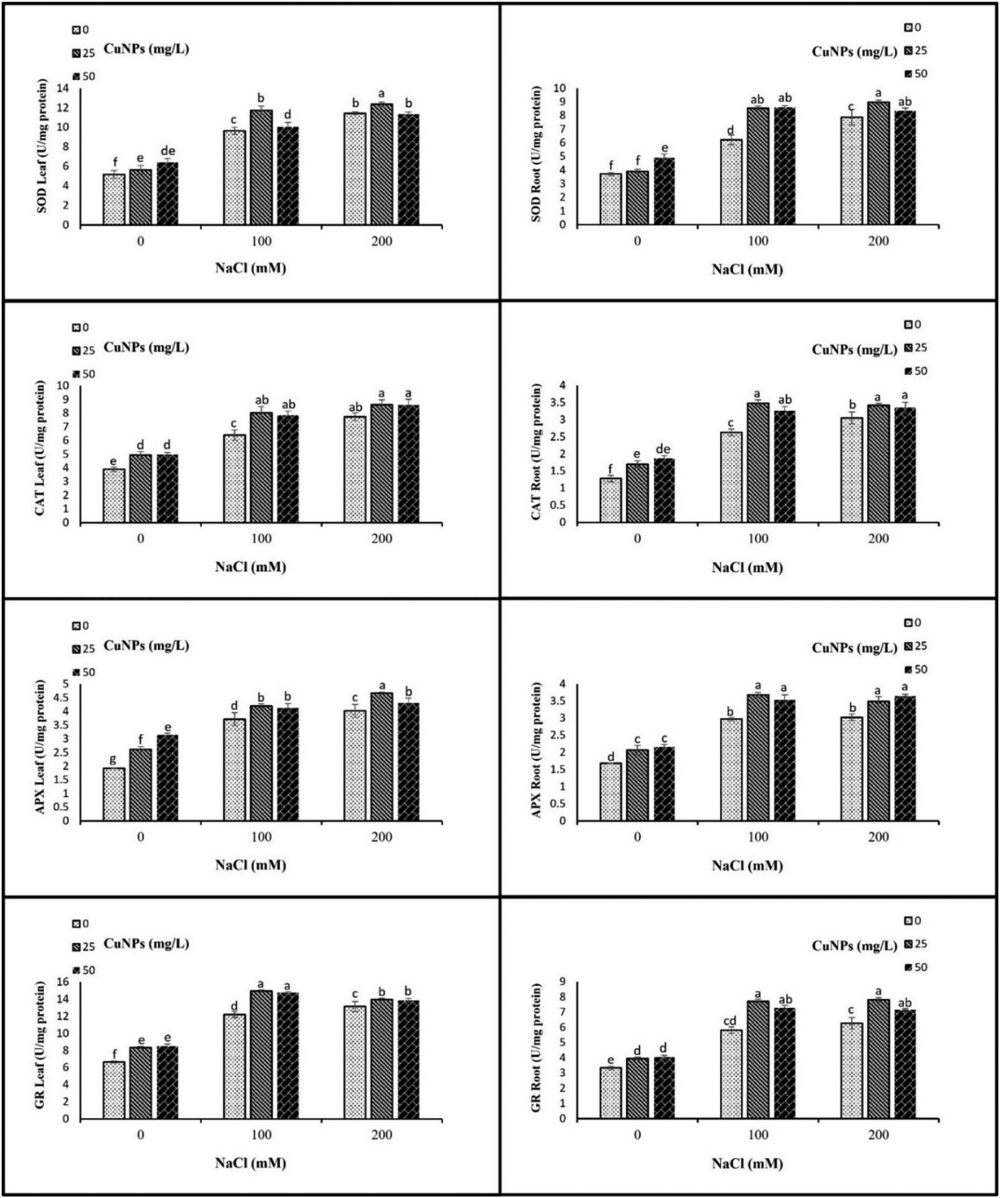
The impact of CuNPs and salt-induced stress, whether applied individually or in conjunction with each other on the antioxidant enzymes activity, (**a**) SOD leaf, (**b**) SOD root, (**c**) CAT leaf, (**d**) CAT root, (**e**) APX leaf, (**f**) APX root, (**g**) GR leaf, and (**h**) GR root in barley (*H. vulgare*). All the data are means of three replicates ± standard deviation. Different letters indicate significant differences between treatments according to Duncan’s multiple range test (p ≤ 0.05).

The present results demonstrate that NaCl treatments at various concentrations led to a significant increase in the activities of enzymatic antioxidants. A treatment of 100 and 200 mM NaCl notably enhanced the activities of SOD, CAT, APX, and GR compared to the control group. The application of CuNPs salinized plants caused more increment in the antioxidant enzyme activities compared with their respective control (only salt-treated plants) in both shoots and roots. These changes were more pronounced with 25 mg/L CuNPs application than 50 mg/L CuNPs application (Fig. 7).

### Nonenzymatic antioxidants

**Figure 8:**
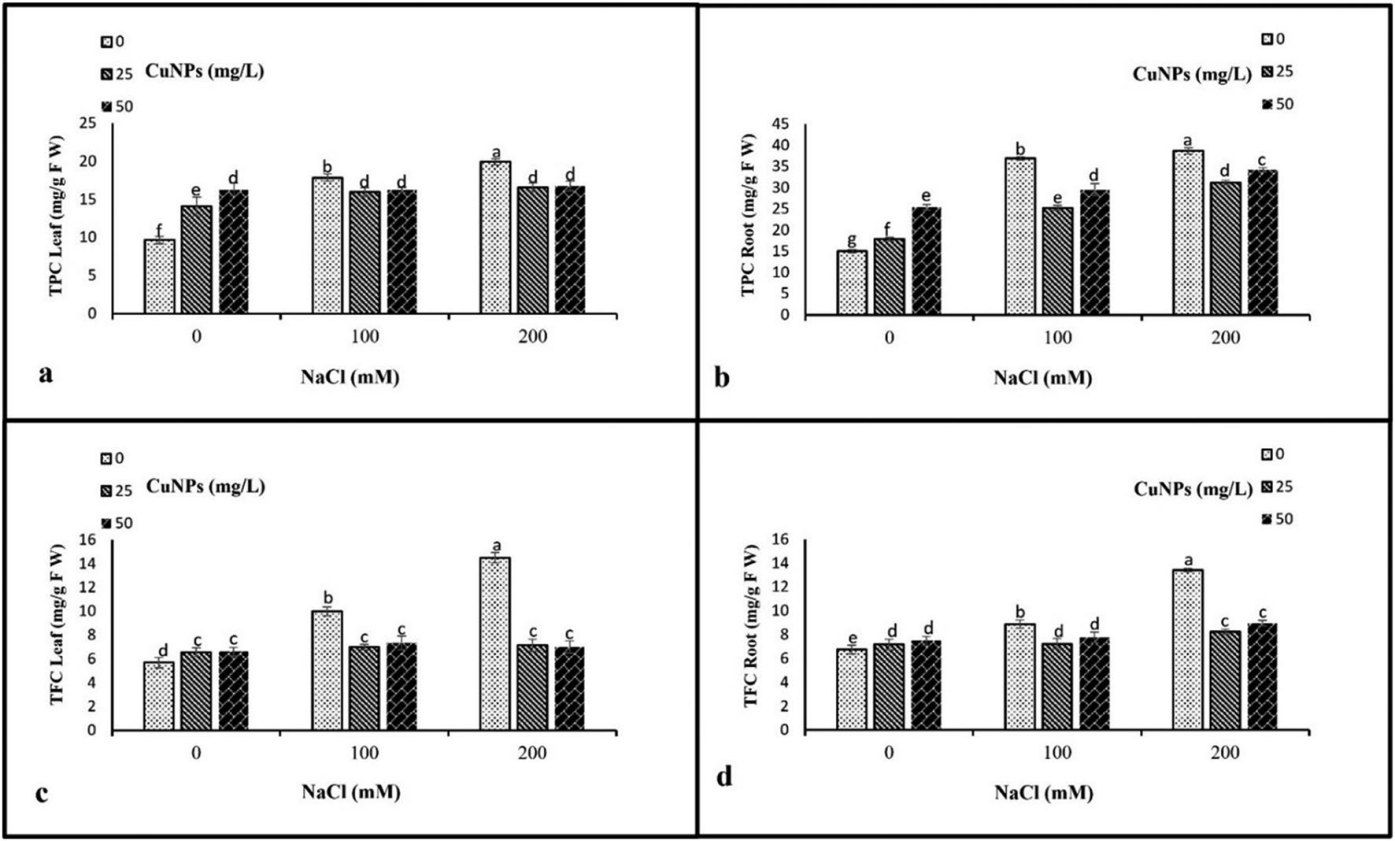
The impact of CuNPs and salt-induced stress, whether applied individually or in conjunction with each other on the non-enzymatic antioxidant compounds, (**a**) TPC leaf, (**b**) TPC root, (**c**) TFC leaf, and (**d**) TFC root in barley (*H. vulgare*). All the data are means of three replicates ± standard deviation. Different letters indicate significant differences between treatments according to Duncan’s multiple range test (p ≤ 0.05).

The nonenzymatic antioxidants, including total phenol (TPC) and total flavonoids (TFC) contents, exhibited a significant increment in plants treated with 100 and 200 mM NaCl in comparison to control plants. The most substantial increase was observed in plants exposed to 200 mM NaCl. Interestingly, the application of 25 mg/L CuNPs in the two salinity levels (100 and 200 mM) significantly reduced the TPC and TFC barley-leaves and roots compared to their respective controls (Fig. 8).

### Correlation study

**Figure 9:**
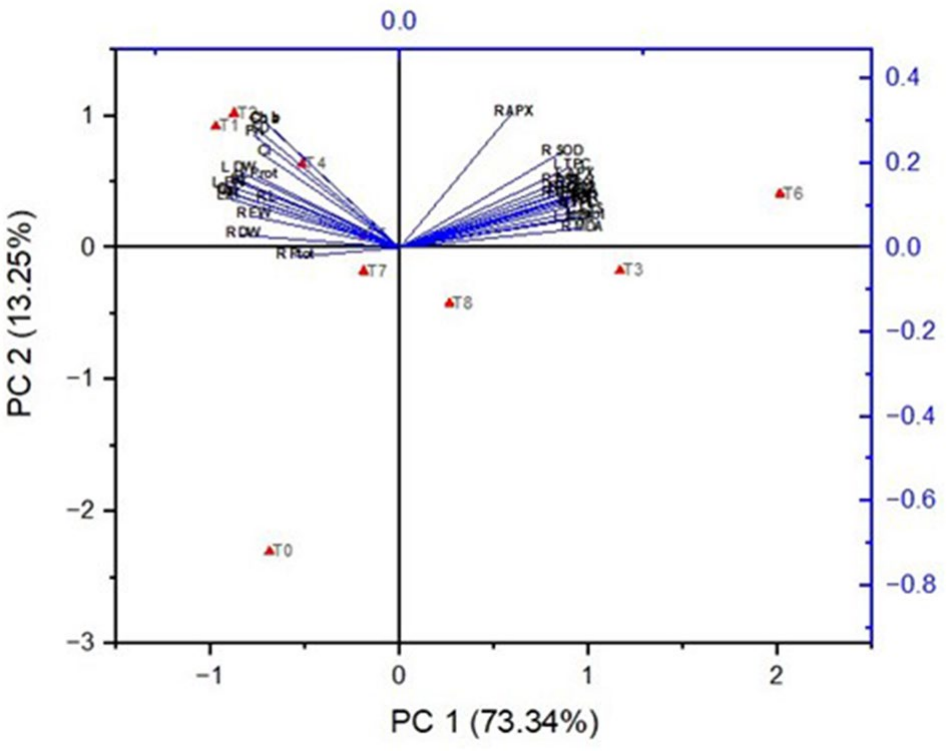
Principal component analysis (PCA) of morpho-physiological characteristics of barley plants exposed to different concentrations salt stress and application of CuNPs.

**Figure 10:**
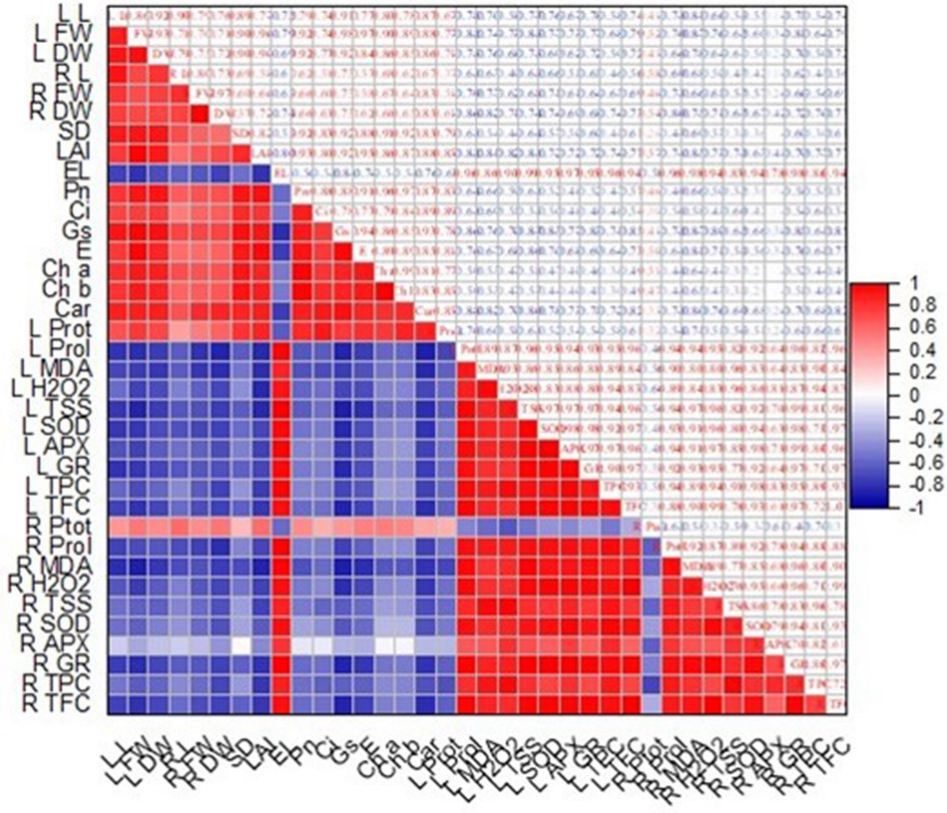
The correlation between different morpho-physiological characteristics of barley plants exposed to different salt stress and application of CuNPs.

PCA and Pearson's correlation analyses were conducted to comprehend the connections between different CuNPs treatments and the diverse morpho-physiological parameters in barley under salinity. Figure 9 presents a PCA biplot which explained 86.59% (73.34% and 13.25%) of the total variance. Furthermore, Pearson’s correlation matrix reveals a significant correlation among the different morpho-physiological characteristics as shown in (Fig. 10). In summary, the growth parameters exhibited positive correlations with fresh weight, dry weight, plant height, chlorophyll, and gas exchange parameters content. Conversely, they showed negative correlations with electrolyte leakage, lipid peroxidation, H_2_O_2_ and proline contents.

## Discussion

Characterization of biosynthesized nanoparticles involves common techniques such as UV, XRD, FTIR spectroscopy, and TEM microscopy. In the current study, the formation of the CuNPs was visually confirmed by the observable change in the color of the reaction mixture, which turned to dark green after the application of heat. The UV–vis spectra serve as an indirect method for verifying and analyzing the bioreduction of CuNPs within the CuSO_4_ solution. The spectrum observed at 320 nm confirmed the presence of surface plasmon resonance (SPR) of the CuNPs and suggests that the particles were dispersed without any signs of aggregation. The peak observed at 3430 cm^−1^ in the FTIR spectrum signifies the stretching of O–H bonds in alcohols and phenols, peak observed at 2098 cm^−1^ corresponding to C=H indicates a robust stretching associated with the alkyl methylene group. The peak at 1650 cm^−1^ signifies the carbonyl groups (C=O), and band at 760 cm^−1^ most likely due to the peroxide formation. The presence of these peaks indicated the existence of flavonoid and phenolic acids in the *S. Argel* leaves extract; thus, the reduction of metal ions and the subsequent formation of nanoparticles may be associated with the presence of flavonoids and phenolic functional groups in the extract. The TEM images demonstrated that the synthesized CuNPs were spherical in morphology, with the average size ranging from 9 to 18 nm.

Nanobiotechnology has significantly broadened the horizons of agricultural research, particularly in enhancing the growth and yield of food crops when faced with challenging environmental conditions. This field has opened innovative avenues for utilizing nanoparticles (NPs) to increase plant resilience and protect them against both biotic and abiotic stresses^50^. Among various abiotic stresses, salt-induced stress poses a severe threat to plants, where, several scientists stated that salt stress dramatically hamper germination, decreases plant growth, photosynthesis, stomatal conductance, root length, plant biomass, and generates osmotic stress by disturbing the ion balance and osmoregulation in plants^51–53^.

The application of the biologically synthesized CuNPs has played a significant role in alleviating salt stress on barley growth and positively improved its profile. Under salt stress conditions (100 and 200 mM NaCl), plant growth was adversely affected as indicated by a reduction in various developmental attributes, including stem diameter, leaf area index, fresh weight, dry weight, and the length of both shoots and roots compared to unstressed plants. Naturally, the hindered growth of plants in saline conditions can be ascribed to the excessive accumulation of sodium (Na^+^) and chloride (Cl^−^) ions within various cell compartments in both the roots and above-ground parts of the plant^8,54^. The accumulation of these ions to mischievous levels disrupts genetic expression, hinders protein synthesis, impairs enzymatic processes, affects energy metabolism, and inhibits cell division; this also results in damage to the structural integrity of the cells, ultimately leading to the possibility of cell death^1,55,56^. However, when copper nanoparticles (CuNPs) were applied to the salt-stressed plants, these detrimental effects were reversed, and a notable improvement in growth characteristics was observed. The enhanced growth of barley plants treated with CuNPs can be attributed to the elevation of cellular antioxidant levels, which in turn, helped alleviate oxidative stress by effectively scavenging ROS^57^. These findings align with previous report by Hernández-Hernández et al.^58^, they found that CuNPs positively affected the growth and physiology of tomato plants under saline stress by controlling oxidative stress.

Regulating the parameters of leaf gas exchange is a crucial factor in enhancing crops resilience to different biotic and abiotic stressors. Our findings showed that the exposure of barley plants to salt stress (100 and 200 mM) caused a drastic reduction in barley leaf gas exchange. The salt-induced decreases in leaf gas exchange parameters, including *Ci*, *Tr*, *gs*, and *Pn*, have been observed in barley plants^59^ and oil palm^60^. Interestingly, CuNPs have been shown to increase the values of barley leaf gas parameters under salt stress in comparison to salinized plants. A similar result was reported by Javeed et al.^61^ who found that the gas exchange parameters were increased after application of zinc oxide nanoparticle in *Lagenaria siceraria L.*

The decrease in photosynthetic pigments in the leaves of barley plants under saline conditions aligns perfectly with the discovery made by Narimani et al.^62^. The reduction in pigment levels was most likely attributed to increased damage of chloroplast structure, instability in pigment-protein complexes, and heightened chlorophyllase activity^63^. Our findings designated a similar decrease in photosynthetic pigments content in barley plants treated with salt stress alone. Interestingly, the application of CuNPs at different concentrations resulted in a considerable increase in photosynthetic pigments in both salt-treated and non-treated barley plants. The highest point of this increase was observed in plants that had been exposed to 25 mg/L CuNPs. Copper nanoparticles enhance nutrient uptake in plants, which can lead to better pigment synthesis and maintenance^64^. This can result in an augmentation of chlorophylls or other supplementary pigments in the plant leaves.

Plants have an inherent ability to boost the synthesis of osmolytes such as soluble protein, soluble sugars and proline within their cytosol and various organelles, which helps them counteract the adverse impact of salt toxicity^65^. In this study, the levels of soluble sugars, soluble proteins, and proline were significantly elevated in barley plants subjected to NaCl stress. Several studies have revealed that salt-induced stress resulted in increased levels of osmoregulatory substances including soluble sugars, soluble proteins, and proline in rice, faba bean and artichoke plants^66–68^. The application of CuNPs resulted in a reduction in the accumulation of total soluble proteins, total soluble sugars and proline in the stressful barley plants compared to their respective control. Our findings align with the results presented in the study by Mohamed et al.^69^, in which they observed that nanoparticles decreased soluble sugar, and proline levels in plants under salinity stress compared to their respective salt-treated plants alone.

The excessive salt accumulation in the cytoplasm leads to hyperosmotic stress and ionic imbalance, hence, triggers the production of ROS like hydrogen H_2_O_2_ as well as overproduction of MDA. In this study, a notable rise in the levels of MDA and H2O2 was observed in barley plants subjected to salt stress alone, aligning with previous findings in soybean^70^ and strawberry^71^, where MDA and H_2_O_2_ concentrations were found to increase in response to salinity. This accumulation most likely attributed to the shock and photo-oxidative stress induced to salinity stress as suggested by^70,72^. Furthermore, salinity causes a drastic reduction in the water status of the stressed plants due to higher accumulation of the sodium ions; this is probably another reason responsible for the increase in H2O2 and MDA concentrations.^73^. The application of CuNPs to salinized plants resulted in a significant reduction in oxidative stress and lipid peroxidation as indicated by the observed decrease in the levels of H_2_O_2_ and MDA .The positive impacts of CuNPs on barley plants in mitigating oxidative damage caused by salinity appear to be linked to the upregulated activity of enzymatic antioxidants by swiftly disposal of H_2_O_2_, thereby promoting and sustaining plant growth^74^.

Numerous studies have indicated that exposure to salt stress can trigger the production of reactive oxygen species, leading to heightened activity of antioxidant enzymes as a protective mechanism^74–76^. This is consistent with our results, where the levels of antioxidant enzymes including SOD, CAT, APX, and GR activity significantly increased in barley plants as response to salt stress. Interestingly, our findings demonstrated that the use of CuNPs led to more enhancement in the activity of antioxidant enzymes in comparison to only salt-stressed plants. The increment in the activities of antioxidant enzymes in the salt-stressed plants after application of NPs suggests that this nanoparticle effectively mitigated the effects of salinity stress on barley plants. In line with our results, the application of CuNPs promoted the activities of enzymatic antioxidants in maize and tomato under salinity conditions^57,77^.

Phenolic and flavonoid compounds are vital for safeguarding plants against various biotic and abiotic stressors. They also function as co-factors for enzymes, influencing the growth and development of plants, starting from their early stages and continuing through senescence^78,79^. Our results in response to salt stress indicate significant enhancements in TPC and TFC compared to control group. The elevations in phenolic contents might be due to higher expression of genes in phenolic biosynthetic pathway^80^, while, increment in flavonoids considered as a part of the adaptive reaction to salt stress^81^. The CuNPs successfully reduced phenols and flavonoid content on barley plants treated with NaCl. This findings in alignment with Hanif et al.^82^ report, who found that NPs caused reduction in TPC and TFC on *Coriandrum sativum* plants under salinity. The reduction in total phenolic and total flavonoid contents indicates that they are needed in lower quantities because of reduced ROS production following the application of copper nanoparticles^9^.

The findings of this research suggest that the negative impacts of salt stress can be reduced by using CuNPs. Thus, these CuNPs, produced through biological synthesis, can be utilized in salt-affected agricultural settings as a cost-effective and environmentally friendly solution to boost crop yield. However, Further research in this area is necessary, with an emphasis on exploring the potential of next-generation technologies, including nanomodified stimulants and nano-based smart sensors, to address agricultural challenges such as salinity stress.

## Conclusion

This study underscores the significant connection between sustainable agricultural practices and nanoscience. It particularly highlights the positive impact of biologically synthesized copper nanoparticles (CuNPs) from *S. argel* leaf extract in mitigating the detrimental effects of salinity stress on various plant responses. We can infer that the application of biologically synthesized CuNPs induced a significant change into barley plants under salinity conditions, where mitigated the adverse effects of NaCl stress by promoting plant growth, improving gas exchange parameters, boosting photosynthetic pigments, regulating osmoregulation, and reducing the accumulation of MDA and H_2_O_2_. Additionally, protection against NaCl-induced stress was achieved by minimizing total phenols, flavonoids, and enhancing the activities of antioxidant enzymes. According to the results, this study concluded that the application of biosynthesized CuNPs to barley plants could serve as a tactic to enhance their salt tolerance, thereby improving plant growth, physiological parameters, and overall yield. However, further research efforts are needed to comprehensively understand how copper nanoparticles alleviate the detrimental impacts of salt in plants at both molecular and genetic levels.

## Data Availability

The datasets generated and analyzed in the current study are available from the corresponding author upon reasonable request.
